# Physical organic chemistry

**DOI:** 10.3762/bjoc.6.116

**Published:** 2010-11-03

**Authors:** John A Murphy

**Affiliations:** 1 WestCHEM, Department of Pure and Applied Chemistry, University of Strathclyde, 295 Cathedral Street, Glasgow G1 1XL, U.K

Physical organic chemistry – the study of the interplay between structure and reactivity in organic molecules – underpins organic chemistry, and we cannot imagine organic chemistry as a subject without knowledge of mechanism and reactivity. It is sometimes thought that the golden age of ‘physical organic chemistry’ was in the 20^th^ century, when systematic information about mechanism first burst onto the scene. Certainly the impact of early knowledge of mechanism of fundamental aliphatic substitution reactions, among others, was enormous, but our knowledge of reactivity and mechanism has continued to progress and deepen enormously ever since and this has been reflected in a number of Nobel Prizes in Chemistry. In an area of particular interest to me, the transformation of radical chemistry from being an almost impenetrable area to one that can be usefully harnessed even in synthetic applications, has been extraordinary – this transformation has been relatively recent and has been principally dependent on the accurate determination of kinetics of radical reactions.

Applications to complex reactions in biology, polymer chemistry and electronic materials are ever more prevalent, and add to contributions in ‘small molecule’ chemistry. Novel experimental techniques combined with the revolution in computational chemistry give new impetus to physical organic chemistry and contribute to its continuing importance, an importance that is reflected in the large number of international meetings in physical organic chemistry in the past two years.

I am privileged to act as Guest Editor for this Thematic Series of the Beilstein Journal of Organic Chemistry, and hope that you enjoy the papers that form this issue. I am grateful to the contributors for their contributions.

John A. Murphy

Glasgow, November 2010

